# Ultrasound-guided minimally invasive interventions for refractory musculoskeletal pain: a real-world study from an interventional rheumatology unit

**DOI:** 10.3389/fmed.2026.1932913

**Published:** 2026-08-31

**Authors:** Maria Beatriz Paredes-Romero, Martina Steiner, Ana Valeria Acosta, Israel J. Thuissard, Ana Isabel Castillo-Varón, Gabriela Cueva-Nájera, Marco Algarra-San José, Ana Esteban-Vázquez, Tatiana Cobo-Ibáñez, María Liz Romero-Bogado, Laura Trives-Folguera, Cristina Vergara-Dangond, Cristina Bernal-Morales, Patricia Richi-Alberti, Isabel De La Cámara, Santiago Muñoz-Fernández

**Affiliations:** 1 Rheumatology Service, Hospital Universitario Infanta Sofía, Madrid, Spain; 2 Faculty Of Medicine, Health and Sports, Universidad Europea De Madrid, Madrid, Spain; 3 Rheumatology Service, Hospital Universitario Infanta Sofía, Infanta Sofía and Henares Hospitals Foundation for Biomedical Research and Innovation (FIIB HUIS HHEN), Madrid, Spain

**Keywords:** erector spinae block, interventional rheumatology, musculoskeletal pain, pain management, real-world evidence, ultrasound-guided procedures

## Abstract

**Background:**

Chronic musculoskeletal pain represents a global health challenge, with a substantial proportion of patients failing to achieve adequate symptom control despite conventional treatment. Interventional rheumatology units (IRUs) incorporating ultrasound-guided minimally invasive procedures have emerged as a promising strategy for the management of refractory musculoskeletal pain. However, real-world evidence regarding the performance of these units in routine clinical practice remains limited. This study evaluated the effectiveness and safety of an IRU in routine clinical practice.

**Methods:**

This retrospective, observational, single-center study included consecutive patients with chronic musculoskeletal pain refractory to conventional treatment, who underwent ultrasound-guided interventions at an IRU between April 2023 and February 2026. Procedures were categorized as myofascial infiltrations, shoulder procedures, erector spinae plane block, and a miscellaneous group of other procedures. The primary outcome was a clinically meaningful pain reduction, defined as a reduction of at least 2 points on the Numerical Rating Scale (NRS) at the first post-procedure follow-up visit.

**Results:**

A total of 170 patients (77.6% women; median age 61 years) were included. Overall, 125 of 170 patients (73.5%) achieved the primary endpoint, with no significant differences across procedural groups. Median NRS decreased from 7.0 [IQR, 7.0–8.0] to 3.0 [IQR, 2.0–6.0] at follow-up, corresponding to a median reduction of 4.0 points (IQR, 1.0–6.0; *p* < 0.001). Two minor adverse events (1.2%) were recorded, and 30 patients (17.6%) required repeat procedures. In multivariable logistic regression analysis, a higher baseline NRS score was the only independent predictor of a clinically meaningful response (OR 1.74, 95% CI, 1.30–2.34; *p* < 0.001). Overall, 50 patients (29.4%) required referral to another specialty, although no baseline clinical variable independently predicted this outcome. Overall, 50 patients (29.4%) required referral to another specialty, although no baseline clinical variable independently predicted this outcome; the remaining 70.6% were managed or discharged by the IRU.

**Conclusion:**

Ultrasound-guided minimally invasive procedures performed within an IRU were associated with high rates of clinically meaningful pain reduction, and a favorable safety profile in patients with chronic musculoskeletal pain. These findings suggest that IRUs may have a role within multidisciplinary pain management pathways, although prospective controlled studies are needed to confirm these results.

## Introduction

Chronic musculoskeletal pain represents a global health challenge, affecting approximately 30% of the adult population and serving as a leading cause of disability worldwide (1). In Spain, chronic pain affects approximately one in four adults, with the highest prevalence among women and adults aged 57–75 years. More than half of patients report high pain intensity, and nearly half of those in active employment require sick leave, placing a substantial economic burden on the healthcare system.

Furthermore, chronic pain markedly impairs quality of life and is frequently associated with social isolation, depression, and reduced mobility (2).

Conventional management of chronic pain typically follows the World Health Organization (WHO) analgesic ladder, which consists of a three-step approach based on increasing analgesic potency (3). However, pharmacological treatment is often inadequate or limited by safety concerns. Acetaminophen has shown limited efficacy for common chronic pain conditions such as low back pain and osteoarthritis, whereas non-steroidal anti-inflammatory drugs (NSAIDs), although effective for many patients, are associated with an increased risk of gastrointestinal, cardiovascular, and renal adverse events, particularly in older adults. Furthermore, the long-term use of opioids remains controversial because evidence supporting sustained efficacy beyond 6 weeks is limited, whereas prolonged exposure is associated with an increased risk of opioid-related harms, including addiction and overdose (4). Consequently, many patients do not achieve adequate pain control with conventional pharmacological therapies, underscoring the need for effective and evidence-based interventional pain management strategies (2).

In this context, the establishment of specialized interventional rheumatology units has become an important strategy to address these therapeutic gaps through targeted, minimally invasive diagnostic and therapeutic procedures (5). The integration of ultrasound guidance represents a major advance in the evolution of this field, providing substantial improvements in both safety and procedural precision (6). Compared with conventional landmark-based techniques, ultrasound enables real-time visualization of relevant anatomical structures, allowing precise needle placement and continuous monitoring of injectate spread (7). These advantages reduce the risk of procedure-related complications, including pneumothorax, inadvertent vascular puncture, and nerve injury (8).

**Figure 1:**
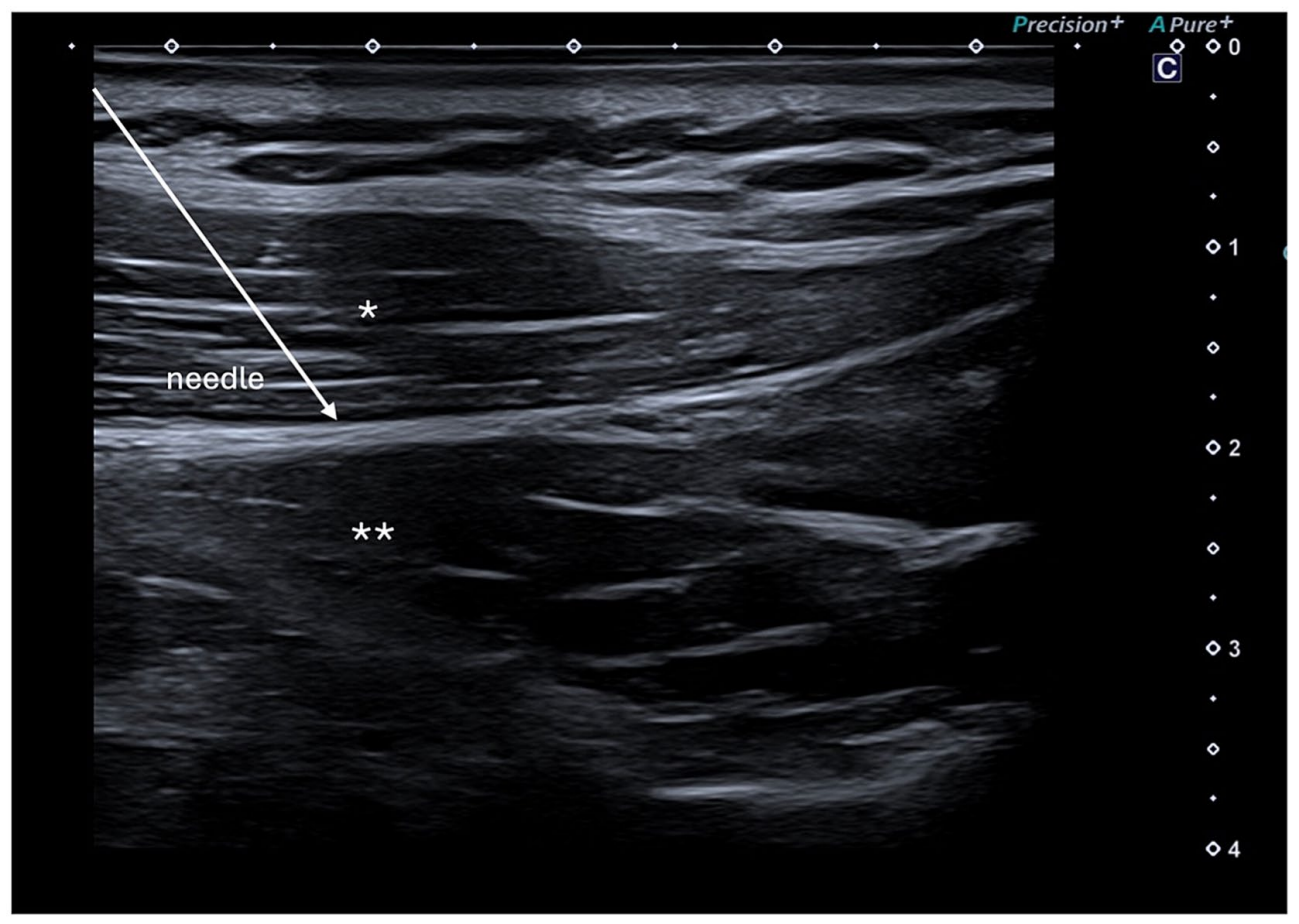
Longitudinal ultrasound image showing fascial plane infiltration. The needle (arrow) is advanced to target the interfascial plane between the Trapezius muscle (*) and the underlying Rhomboid muscle/Levator Scapulae muscle (**).

**Figure 2:**
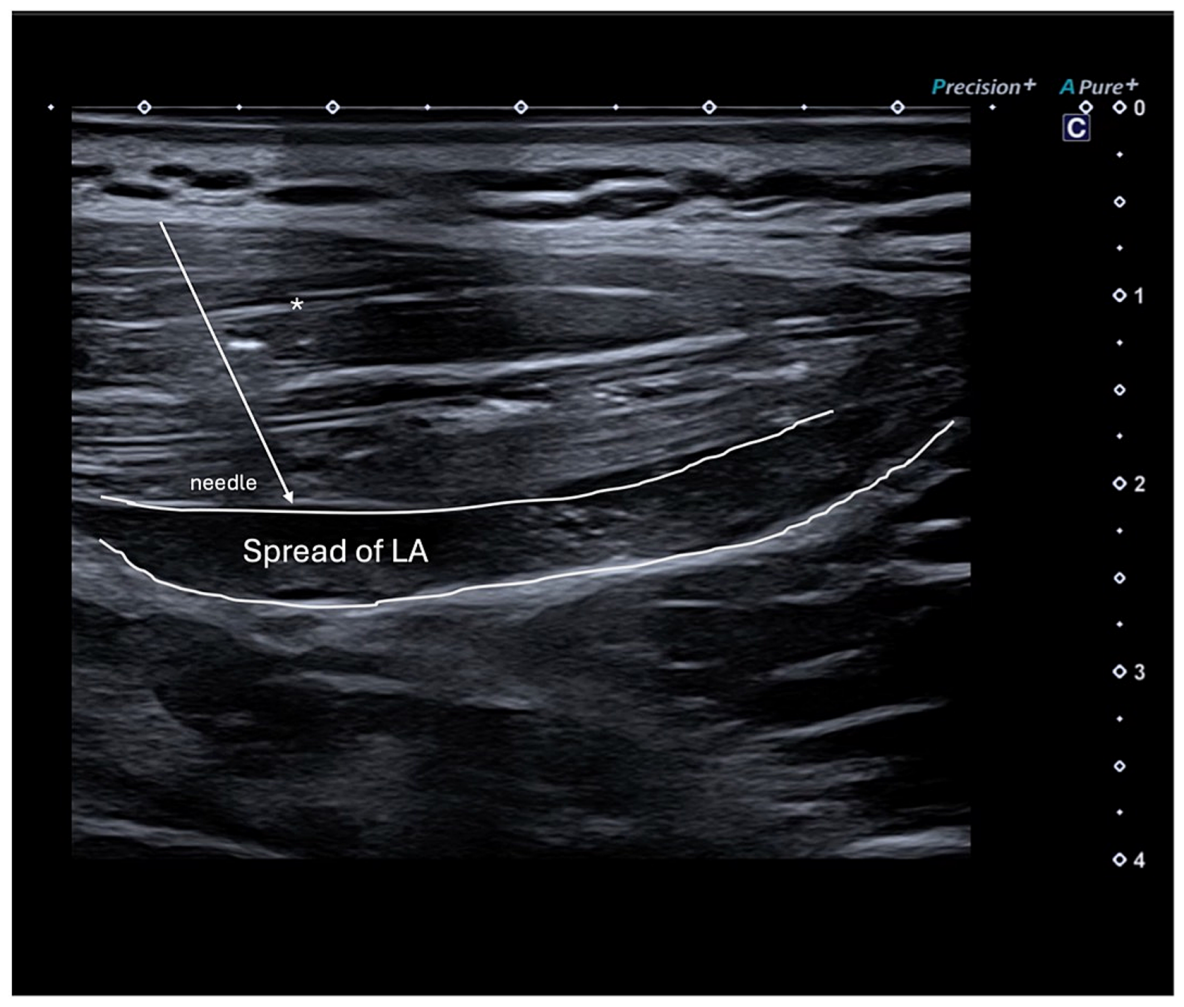
Longitudinal ultrasound image demonstrating hydrodissection of the interfascial plane between the trapezius muscle (*) and the underlying rhomboid/levator scapulae musculature. The needle (arrow) is seen within the plane, with anechoic spread local anesthetic (LA) of the injectate (outlined) distending the fascial space.

An important group of procedures in interventional rheumatology targets myofascial pain syndrome, a condition characterized by myofascial trigger points, which are hypersensitive foci located within taut bands of skeletal muscle (9). Procedures such as trigger point injections and piriformis muscle injections aim to disrupt the pain cycle by mechanically inactivating trigger points and delivering local anesthetics, with or without corticosteroids (9–11). Trapezius fascial plane injections (Figures 1, 2) and quadratus lumborum interfascial injections provide broader analgesic coverage by promoting diffusion of the injectate within fascial planes (9, 12). Ultrasound guidance enhances the accuracy of these procedures, particularly in deep muscles such as the piriformis and quadratus lumborum, where landmark-based techniques are associated with a greater risk of inaccurate needle placement and injury to adjacent neurovascular structures.

**Figure 3:**
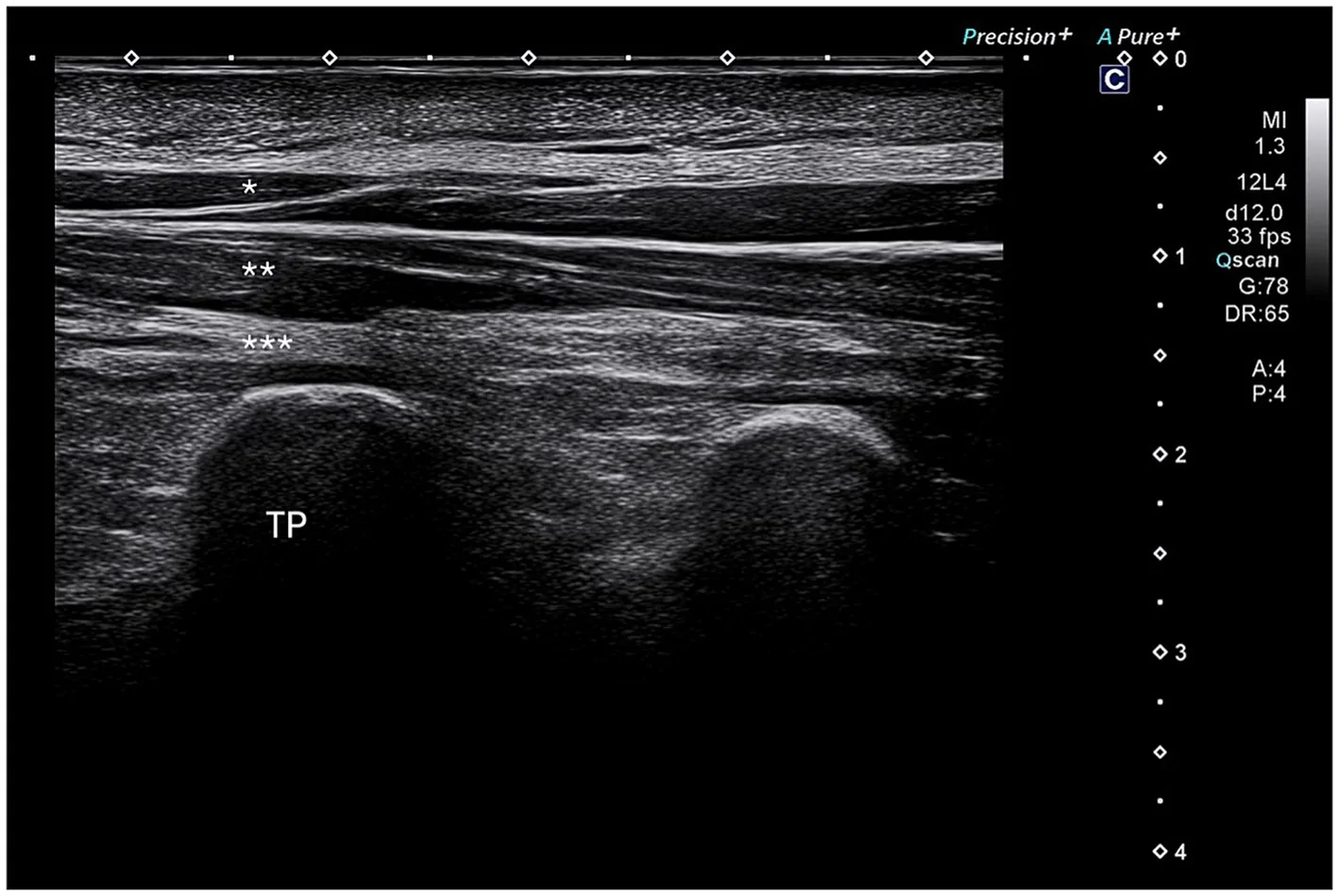
Longitudinal ultrasound image (linear transducer) showing the layered paraspinal anatomy at the thoracic level. From superficial to deep: trapezius muscle (*), rhomboid muscle (**), and erector spinae muscle (***), overlying the transverse process (TP), visible as a hyperechoic bony landmark with posterior acoustic shadowing.

**Figure 4:**
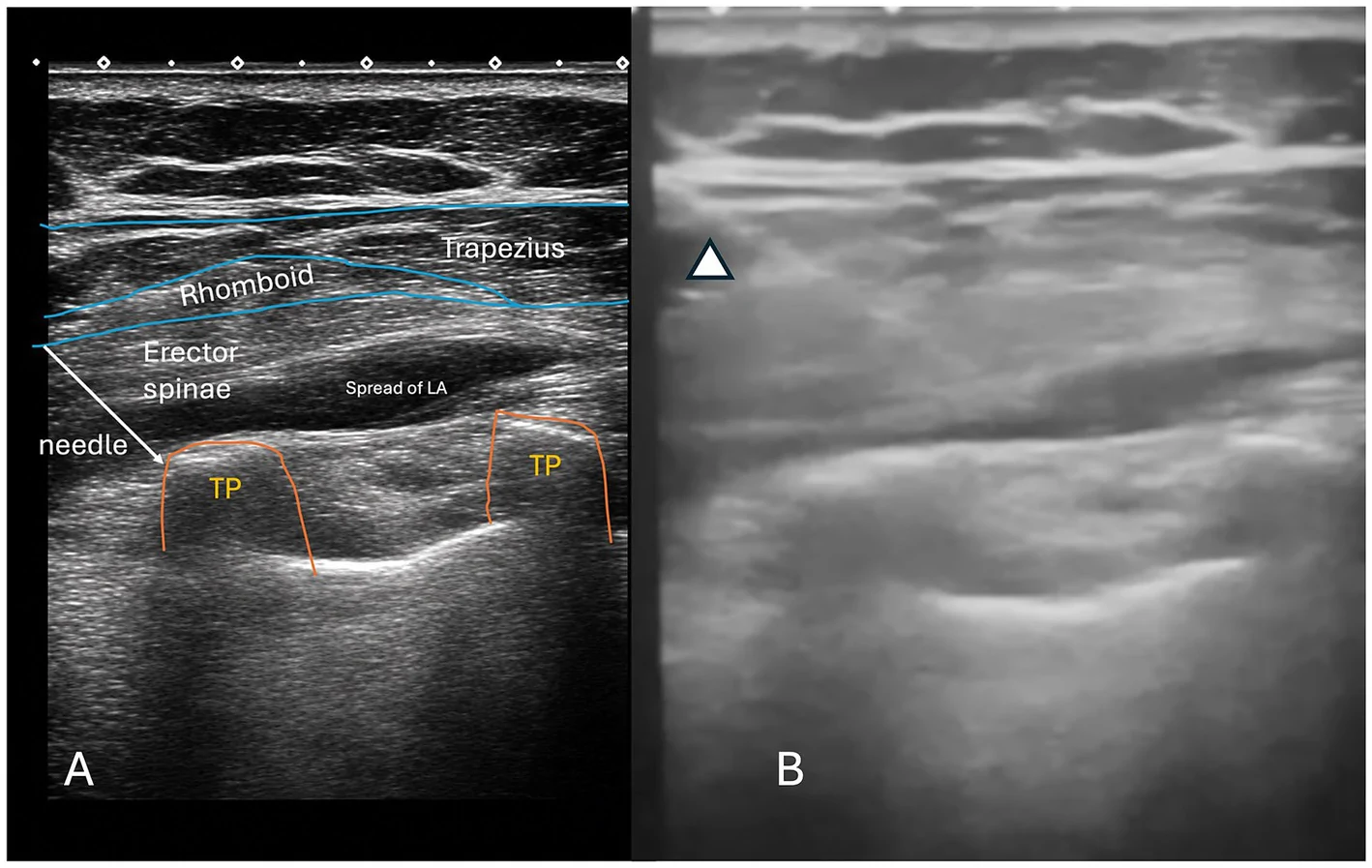
Ultrasound-guided erector spinae plane (ESP) block. **(A)** Longitudinal paravertebral ultrasound view demonstrating needle placement in the fascial plane deep to the erector spinae muscle and superficial to the transverse processes (TP). The spread of local anesthetic (LA) is visible as an anechoic area separating the erector spinae muscle from the transverse processes, producing the characteristic cephalocaudal spread across multiple spinal segments. Trapezius and rhomboid muscles are identified superficially. **(B)** Longitudinal ultrasound image demonstrating the needle (triangle) within the erector spinae interfascial plane. Although the visible portion of the needle has shifted slightly because of probe or needle repositioning during the procedure, the needle tip remains correctly positioned within the interfascial plane.

For axial and thoracic pain, the erector spinae plane (ESP) block has become an increasingly used ultrasound-guided technique since its first description in 2016 (13, 14). The procedure involves injection of local anesthetic into the fascial plane deep to the erector spinae muscle and superficial to the transverse processes, resulting in cephalocaudal spread across multiple spinal levels and blockade of the dorsal and ventral rami (Figures 3, 4) (14). Emerging evidence suggests that ESP block provides effective analgesia in patients with osteoporotic vertebral compressive fractures, reducing pain intensity and opioid requirements while facilitating early rehabilitation (15).

**Figure 5:**
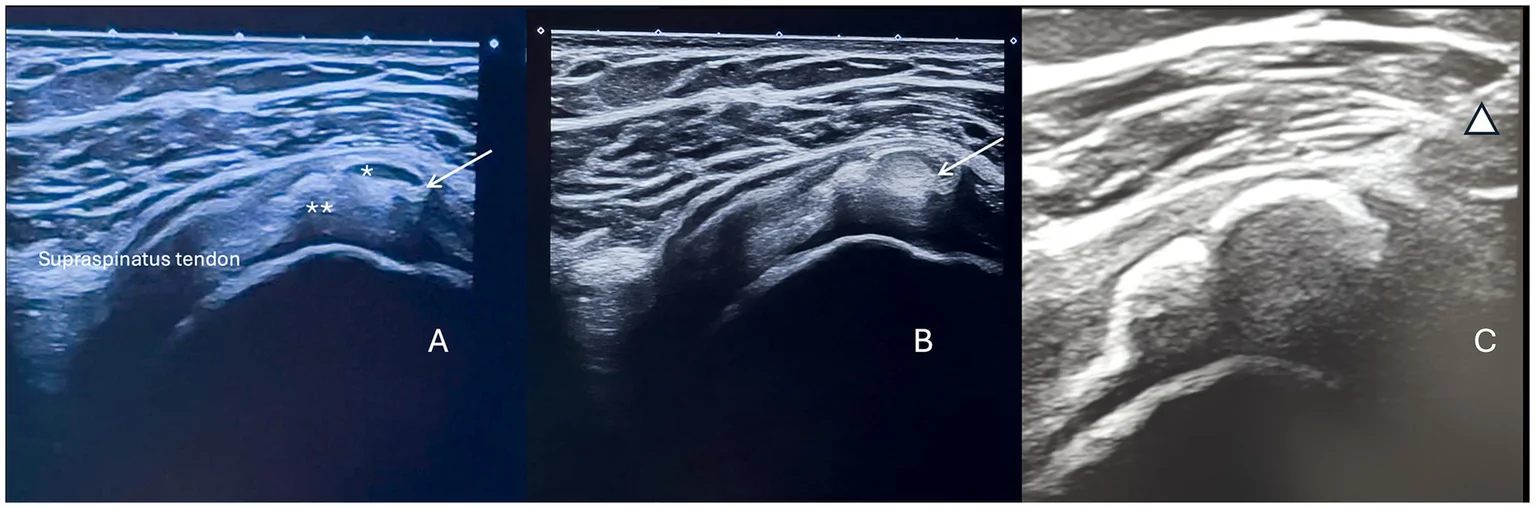
Ultrasound-guided calcific deposit lavage (barbotage) of the supraspinatus tendon. Sequential ultrasound images during the procedure. **(A)** Needle placement (arrow) within the calcific deposit (**) with saline solution (*) injected under real-time visualization; the supraspinatus tendon is identified. **(B)** Active calcium removal showing fragmentation and mobilization of the hydroxyapatite deposit during lavage (arrow). **(C)** The triangle indicates the needle, visualized as a hyperechoic linear structure adjacent to the calcific deposit.

Another major group of interventional procedures focuses on the shoulder, reflecting the high prevalence of shoulder pain in clinical practice (6). These procedures include suprascapular nerve blocks, ultrasound-guided injections, and ultrasound-guided barbotage for calcific tendinopathy (16). Suprascapular nerve blocks have been shown to be safe and effective for chronic shoulder pain and functional impairment, providing symptomatic relief in conditions such as adhesive capsulitis and rotator cuff tendinopathy. In patients with calcific tendinopathy, ultrasound-guided barbotage enables precise fragmentation and aspiration of hydroxyapatite deposits under real-time visualization (Figure 5), leading to significant improvements in pain and functional outcomes while reducing the need for surgical intervention (17, 18).

Despite the growing body of evidence supporting individual ultrasound-guided interventions, data evaluating the overall clinical activity of IRUs in routine practice remain limited. Most published studies have focused on single procedures or specific clinical indications. Therefore, the aim of this study was to evaluate the effectiveness and safety of ultrasound-guided minimally invasive procedures performed at an IRU in patients with chronic musculoskeletal pain refractory to conventional treatment.

## Methods

### Study design and setting

This retrospective, observational, single-center study was conducted at the IRU of Hospital Universitario Infanta Sofía (Madrid, Spain). The IRU was established in April 2023 to provide ultrasound-guided minimally invasive procedures for the management of refractory musculoskeletal pain.

The study was approved by the Ethics Committee for Clinical Research with Medicines (CEIm) of the Hospital Universitario de Getafe (Madrid, Spain) on February 26, 2026 (ref. CEIm36/32). The study was conducted in accordance with the Declaration of Helsinki, the Belmont Report, and applicable Spanish and European data protection regulations (Organic Law 3/2018 and EU Regulation 2016/679).

### Participants

All consecutive patients who underwent at least one ultrasound-guided procedure at the IRU between April 2023 and February 2026 were considered eligible for inclusion.

Eligible patients were required to meet the following inclusion criteria: age ≥18 years at the time of the procedure and chronic musculoskeletal pain refractory to conventional treatment, defined as pain persisting for at least 3 months despite adequate trials of conventional management, including pharmacological therapy and/or physiotherapy, with or without previous locoregional infiltrations performed in routine clinical practice. Additionally, eligibility required the availability of complete records of all procedures performed at the IRU, including the date and type of each procedure, as well as at least one post-procedure follow-up visit. Pain intensity had to be assessed at baseline and during follow-up using the Numeric Rating Scale (NRS; 0–10), and these data were required to be available for all included patients.

Exclusion criteria comprised procedures performed outside the IRU; duplicate records (for which the most complete record was retained), and the absence of post-procedure clinical follow-up.

### Procedures

All procedures were performed by two rheumatologists with more than 10 years of experience in ultrasound-guided techniques. Both rheumatologists had completed dedicated training in interventional rheumatology at the IRU of Hospital Universitario de Gran Canaria Dr. Negrín (Las Palmas de Gran Canaria, Spain). Procedures were performed using high-resolution musculoskeletal ultrasound systems equipped with 4–12 MHz multifrequency linear-array transducers.

Patients were classified into four procedural groups according to the type of intervention performed: (I) shoulder procedures, including shoulder injections, suprascapular nerve blocks, and ultrasound-guided barbotage for calcific tendinopathy; (II) myofascial procedures, including trigger point injections, trapezius fascial plane injections, piriformis muscle injections, and interfascial injection at the posterior quadratus lumborum plane; (III) erector spinae plane (ESP) block; and (IV) a miscellaneous group of other procedures.

The pharmacological agents used were triamcinolone acetonide or betamethasone as corticosteroids, ropivacaine as the local anesthetic, and isotonic saline (0.9% NaCl) which was used for calcific deposit barbotage and to increase injectate volume in selected myofascial procedures. For most procedures, injected volumes varied according to the target anatomical structure and procedure type, typically consisting of 1–2 mL of corticosteroid combined with 3–8 mL of ropivacaine. Needle selection was based on target depth and patient body habitus. A 21-gauge intramuscular needle was used for superficial structures, whereas a 22-gauge spinal needle was preferred for deeper targets or patients with increased subcutaneous thickness.

For myofascial procedures, the needle advanced under real-time ultrasound guidance to the target muscle or fascial plane. Trapezius and quadratus lumborum interfascial injections targeted the fascial plane adjacent to the muscle, with correct needle placement confirmed by real-time ultrasound visualization of injectate spread. For shoulder procedures, correct needle placement was similarly confirmed by visualization of injectate spread within the target structures. Ultrasound-guided barbotage for calcific tendinopathy was performed using a single-needle technique with saline irrigation and aspiration of the calcific material under continuous ultrasound guidance.

For ESP block, the needle was advanced until contact with the transverse process, and the injectate was deposited in the fascial plane deep to the erector spinae muscle. Before injection, negative aspiration was performed to exclude intravascular placement. Correct needle positioning was confirmed by administering 2–3 mL of isotonic saline (0.9% NaCl), visualized as anechoic separation between the erector spinae muscle and the transverse process. For unilateral ESP blocks, the injectate consisted of 6 mL of ropivacaine 0.2%, 1 mL of betamethasone (3 mg), and 8 mL of isotonic saline (total volume 15 mL). For bilateral ESP blocks, 4 mL of ropivacaine 0.2%, 1 mL of betamethasone (3 mg), and 10 mL of isotonic saline were administered on each side.

### Outcomes

The primary outcome was the proportion of patients achieving a clinically meaningful pain reduction, defined as a reduction of ≥2 points on the NRS from baseline to the first post-procedure follow-up visit.

Secondary outcomes included the absolute change in NRS score from baseline to the first follow-up visit, response rates across procedural groups, the need for repeat procedures, procedure-related complications, referrals to other specialties, and predictors of clinical response.

### Data collection

Clinical data were extracted from the electronic medical record system (SELENE) and pseudonymized prior to analysis. To minimize selection bias, all consecutive eligible patients treated during the study period were included.

Data were managed using REDCap (Research Electronic Data Capture), on a secure institutional server with restricted access.

### Statistical analysis

A descriptive analysis was performed for all variables. Categorical variables were summarized as absolute and relative frequencies; 95% confidence intervals were calculated for the primary outcome. Continuous variables were reported as mean ± standard deviation or median with interquartile range [IQR] depending on the distribution of the data, as assessed using the Shapiro–Wilk test.

Baseline and post-procedure NRS scores were compared using the Wilcoxon signed-rank test for paired data. Between-group comparisons of categorical variables were performed using Pearson’s chi-square test or Fisher’s exact test, as appropriate. To identify independent predictors of clinical response, univariable and multivariable logistic regression analyses were performed. The following variables were included as candidate predictors in the univariable logistic regression analysis: age, sex, body mass index (BMI), pain duration, prior infiltration, and procedural group. Variables with *p* < 0.10 in the univariable analysis were entered into the multivariable model. Results are presented as odds ratios (ORs) with 95% confidence intervals (CIs). All statistical tests were two-sided, and statistical significance was defined as *p* < 0.05. Analyses were performed using IBM SPSS statistics version 29.0 (IBM Corp., Armonk, NY, United States).

No formal sample size calculation was performed because this was a retrospective observational study including all consecutive eligible patients treated during the study period. Only patients with complete baseline and follow-up NRS data were included in the analysis; therefore, no imputation of missing data was required.

This study was reported in accordance with the Strengthening the Reporting of Observational Studies in Epidemiology (STROBE) guidelines.

## Results

### Study population

During the study period, 170 consecutive patients met the eligibility criteria and were included in the analysis. No patients were excluded due to missing NRS data, as complete baseline and follow-up pain assessments were available for all included patients.

The cohort was predominantly female (132/170, 77.6%), with a median age of 61 years [IQR, 49.0–73.2]. The median baseline NRS score was 7.0 [IQR, 7.0–8.0], and the median duration of pain prior to the procedure was 12.0 months [IQR, 5.0–24.0]. Median follow-up was 89.5 days [IQR, 53.0–136.0]. Regarding baseline analgesic treatment, 102 patients (60.0%) were at WHO analgesic ladder step 1, 59 (34.7%) on step 2, and 9 (5.3%) on step 3.

Patients were classified into four procedural groups: (I) myofascial infiltrations (*n* = 68, 40.0%), (II) shoulder procedures (*n* = 54, 31.8%), (III) ESP block (*n* = 27, 15.9%), and a miscellaneous procedures group (*n* = 21, 12.4%), predominantly comprising patients with osteoarthritis (15/21, 71.4%). The ESP group had the highest median age (72.7 years; IQR, 58.5–76.1) and the highest proportion of female patients (25/27, 92.6%), consistent with the predominance of osteoporotic vertebral fractures in this group (18/27, 66.6%). Baseline characteristics by procedural group are summarized in Table 1.

**Table 1:** Baseline characteristics by procedural group.

	Overall (*n* = 170)	Shoulder (*n* = 54)	Myofascial (*n* = 68)	ESP (*n* = 27)	Other (*n* = 21)	*p*
Baseline characteristics						
Age (years)	61.0 [49.0–73.2]	57.3 [47.9–66.9]	58.6 [47.9–71.8]	72.7 [58.5–76.1]	61.2 [51.2–73.2]	0.029*
BMI (kg/m^2^)	27.1 [23.5–30.4]	28.0 [24.4–30.9]	27.3 [22.6–30.4]	25.9 [24.0–27.2]	25.9 [23.1–33.5]	0.510
Pain duration (months)	12.0 [5.0–24.0]	12.0 [5.2–24.0]	8.0 [4.0–24.0]	12.0 [6.0–13.0]	10.0 [6.0–14.0]	0.877
Baseline NRS (0–10)	7.0 [7.0–8.0]	7.0 [7.0–8.8]	7.0 [7.0–8.0]	7.0 [7.0–9.0]	7.0 [6.0–8.0]	0.156
Follow-up (days)	89.5 [53.5–136.0]	62.0 [41.2–102.0]	99.5 [59.8–137.2]	90.0 [57.0–132.0]	109.0 [82.0–186.0]	0.007*
Female sex	132/170 (77.6)	34/54 (63.0)	55/68 (80.9)	25/27 (92.6)	18/21 (85.7)	0.010*
Prior infiltration	41/170 (24.1)	33/54 (61.1)	2/68 (2.9)	1/27 (3.7)	5/21 (23.8)	<0.001*
Prior analgesic treatment — WHO ladder, *n* (%)						
Step 1 (non-opioid)	102/170 (60.0)	43/54 (79.6)	36/68 (52.9)	8/27 (29.6)	15/21 (71.4)	<0.001*
Step 2 (weak opioids)	59/170 (34.7)	10/54 (18.5)	29/68 (42.6)	14/27 (51.9)	6/21 (28.6)	–
Step 3 (strong opioids)	9/170 (5.3)	1/54 (1.9)	3/68 (4.4)	5/27 (18.5)	0/21 (0.0)	–
Outcomes						
Response ≥2-point NRS	125/170 (73.5)	42/54 (77.8)	51/68 (75.0)	20/27 (74.1)	12/21 (57.1)	0.324
Post-procedure NRS	3.0 [2.0–6.0]	2.0 [2.0–5.8]	3.0 [2.0–5.2]	3.0 [2.0–6.0]	5.0 [2.0–6.0]	0.573
NRS reduction	4.0 [1.0–6.0]	5.0 [2.2–6.0]	4.0 [1.8–6.0]	4.0 [1.8–6.0]	2.0 [0.0–6.0]	0.161
Repeat procedure required	30/170 (17.6)	13/54 (24.1)	11/68 (16.2)	5/27 (18.5)	1/21 (4.8)	0.256
Complications (subcutaneous atrophy)	2/170 (1.2)	1/54 (1.9)	1/68 (1.5)	0/27 (0.0)	0/21 (0.0)	–
Specialist referral required	50/170 (29.4)	17/54 (31.5)	18/68 (26.5)	5/27 (18.5)	10/21 (47.6)	0.152
Reason for discharge						
Clinical improvement	95/170 (55.9)	34/54 (63.0)	40/68 (58.8)	14/27 (51.9)	7/21 (33.3)	0.033*
Referral to another specialty	50/170 (29.4)	17/54 (31.5)	18/68 (26.5)	5/27 (18.5)	10/21 (47.6)	–
Ongoing follow-up	25/170 (14.7)	3/54 (5.6)	10/68 (14.7)	8/27 (29.6)	4/21 (19.0)	–

Median [IQR] for continuous variables; *n* (%) for categorical variables. * *p* < 0.05. NRS (numeric rating scale).

### Primary outcome

Overall, 125/170 patients (73.5%) achieved a clinically meaningful pain reduction of ≥2 points on the NRS at the first post-procedure follow-up visit. Clinically meaningful pain reduction rates were: shoulder procedures 77.8%, myofascial infiltrations 75.0%, ESP block 74.1, and 57.1% in the miscellaneous procedures group. No statistically significant differences in response rates were observed across groups (*p* = 0.324). Baseline and post-procedure NRS analysis confirmed statistically significant pain reductions within all four groups.

### Pain outcomes

A statistically significant reduction in NRS scores was observed across all procedural groups following treatment (Table 2). Overall, the median NRS decreased from 7.0 [IQR, 7.0–8.0] at baseline to 3.0 [IQR, 2.0–6.0] at the first follow-up visit, corresponding to a median reduction of 4.0 points (IQR, 1.0–6.0; *p* < 0.001). The greatest absolute reduction in NRS score was observed in the shoulder group [median reduction, 5.0 points (IQR, 2.2–6.0); *p* < 0.001], followed by the myofascial group [4.0 points (IQR, 1.8–6.0); *p* < 0.001] and ESP block group [4.0 points (IQR, 1.8–6.0); *p* < 0.001]. Although the miscellaneous group showed the smallest reduction in NRS score, the reduction remained statistically significant [median reduction, 2.0 points (IQR, 0.0–6.0); *p* = 0.002].

**Table 2:** Baseline and post-procedure NRS analysis - Wilcoxon signed-rank test median [IQR].

Group	*n*	Baseline NRS median [IQR]	Post-procedure NRS median [IQR]	NRS reduction median [IQR]	*p* (Wilcoxon)
Overall	170	7.0 [7.0–8.0]	3.0 [2.0–6.0]	4.0 [1.0–6.0]	<0.001
Shoulder	54	7.0 [7.0–8.8]	2.0 [2.0–5.8]	5.0 [2.2–6.0]	<0.001
Myofascial	68	7.0 [7.0–8.0]	3.0 [2.0–5.2]	4.0 [1.8–6.0]	<0.001
ESP	27	7.0 [7.0–9.0]	3.0 [2.0–6.0]	4.0 [1.8–6.0]	<0.001
Other	21	7.0 [6.0–8.0]	5.0 [2.0–6.0]	2.0 [0.0–6.0]	0.002

Wilcoxon signed-rank test for paired data. NRS (Numeric rating scale), ESP (erector spinae block).

Given the substantial variability in follow-up duration across patients (range 10–383 days), a *post hoc* analysis was performed to assess whether the timing of outcome assessment influenced treatment response. Although a numerical decrease in NRS reduction and response rates was observed with increasing follow-up duration, these differences were not statistically significant (Supplementary Table 1).

### Safety, repeat procedures, and clinical discharge

Two adverse events were recorded during the study period; both were cases of subcutaneous atrophy secondary to corticosteroid injection (2/170, 1.2%). Repeat procedures were required in 30 of 170 patients (17.6%), with no significant differences between procedural groups (*p* = 0.256).

At the end of follow-up, 95 of 170 patients (55.9%) had been discharged because of clinical improvement, 50 (29.4%) had been referred to another specialty, and 25 (14.7%) remained under active follow-up at the time of data collection.

### Referrals to other specialties

Overall, 50 of 170 patients (29.4%) required referral to another specialty, with no statistically significant differences between procedural groups (*p* = 0.152). Referrals were made to the Pain Unit (20/50, 40%), Rehabilitation (19/50, 38.0%), Orthopedic Surgery (8/50, 16.0%), and Neurosurgery (3/50, 6.0%).

The pattern of referral varied across procedural groups. Patients undergoing shoulder procedures were referred predominantly to Rehabilitation (64.7%), whereas those in the myofascial infiltration and ESP block groups were more frequently directed to the Pain Unit (61.1 and 80.0%, respectively). Patients in the miscellaneous procedures group were most frequently referred to Orthopedic Surgery (50.0%).

Patients who required referral to another specialty had a longer median pain duration before the procedure than those who did not require referral [12.0 months (IQR, 7.0–24.0) vs. 8.0 months (IQR, 4.0–15.0); *p* = 0.010]. However, in the multivariable logistic regression analyses, no baseline clinical variable was independently associated with the need for specialist referral.

### Predictors of clinical response

In the univariable logistic regression analysis, a higher baseline NRS score was the only variable associated with increased odds of achieving a clinically meaningful pain response (OR 1.77, 95% CI 1.32–2.38; *p* < 0.001). In the multivariable model, baseline NRS score remained the only independent predictor (OR 1.74, 95% CI 1.30–2.34; *p* < 0.001). No significant associations were observed between treatment response and age, sex, BMI, pain duration, previous infiltration, or procedural group (Table 3).

**Table 3:** Univariable logistic regression-predictors of clinical response (≥2-point NRS reduction).

Variable	OR	95% CI lower	95% CI upper	*p*
Age (years)	1.02	0.99	1.04	0.194
Female sex	0.83	0.36	1.92	0.659
BMI (kg/m^2^)	0.97	0.90	1.04	0.389
Baseline NRS (0–10)	1.77	1.32	2.38	**<0.001**
Pain duration (months)	0.99	0.98	1.00	0.157
Prior infiltration (yes/no)	1.06	0.67	1.67	0.798

Dependent variable: Clinically meaningful response ≥2-point NRS reduction (yes/no). *p < 0.05. NRS (numeric rating scale).

## Discussion

This real-world retrospective study evaluating 170 consecutive patients treated at an IRU demonstrates that ultrasound-guided minimally invasive procedures were associated with clinically meaningful pain improvement and a reassuring safety profile across a heterogeneous population with chronic musculoskeletal pain refractory to conventional treatment. Nearly three in four patients achieved a clinically meaningful pain reduction of ≥2 points on the NRS, with a median absolute reduction of 4 points, and only two minor adverse events were recorded throughout the entire study period. These findings were consistent across all procedural groups, supporting the role of the IRU in delivering ultrasound-guided minimally invasive procedures in routine clinical practice.

### Effectiveness and comparison with existing literature

Given the substantial heterogeneity in indications, underlying pathophysiological mechanisms, and expected duration of benefit across the procedures included in this study, the findings should be interpreted primarily as an evaluation of the overall performance of the IRU as a model of care rather than as an assessment of the efficacy of individual interventional techniques.

The overall response rate of 73.5% observed in this cohort is consistent with those reported for individual ultrasound-guided interventional procedures. In patients with calcific tendinopathy of the shoulder, ultrasound-guided barbotage has been associated with significant pain reduction and functional improvement in 70–80% of patients (18, 19). Similarly, in myofascial pain syndromes, ultrasound-guided trigger point injections and quadratus lumborum infiltration have demonstrated response rates ranging from 60 to 80% in observational studies (10, 20).

For the ESP block in patients with osteoporotic vertebral fractures, Ma et al. reported a mean reduction of approximately 3 points on the NRS after four procedures (15). Although direct comparison should be interpreted with caution, the median pain reduction observed in our ESP block group after a single procedure was of similar magnitude (4.0 points), despite this being the oldest and potentially most therapeutically challenging subgroup in the cohort.

Although cross-group comparisons are limited by differences in patient populations and underlying diagnoses, the consistently high response rates across procedural groups support the feasibility of integrating diverse ultrasound-guided interventions within a single IRU.

### Baseline pain intensity as a predictor of response

The only independent predictor of clinically meaningful pain response identified in the multivariable logistic regression model was baseline NRS score. One plausible explanation is that patients with greater baseline pain have a larger margin for absolute improvement and are therefore more likely to achieve the predefined threshold of ≥2 points reduction on the NRS.

This interpretation is consistent with the work of Farrar et al., who analyzed data from 2,724 patients enrolled in 10 randomized controlled trials and demonstrated that a reduction of ≥2 points on the NRS corresponds to a clinically important improvement from the patient’s perspective. They also showed that patients with higher baseline pain require larger absolute reductions to achieve a similar level of perceived improvement. Therefore, floor effects limit the likelihood of detecting a clinically meaningful response among patients with lower baseline NRS scores, which may partly explain the association observed in our cohort (21, 22).

No demographic variable, including age, sex, or BMI, was independently associated with response. Although these findings should be interpreted in the context of the study design, they suggest that the effectiveness of ultrasound-guided interventional procedures may not be confined to a specific demographic profile. Consequently, demographic characteristics alone may have limited value for identifying patients most likely to benefit from these interventions. In contrast, baseline pain intensity emerged as the main predictor of clinically meaningful pain improvement in this cohort.

These findings differ from those reported by Yildirim Uslu et al., who identified a negative association between age and BMI and treatment response following ultrasound-guided quadratus lumborum block for acute-subacute lumbar strain (20). However, direct comparison between the two studies is limited by substantial differences in patient population, pain chronicity, and procedural protocols.

Several methodological differences may explain these discrepant findings. First, follow-up in the study by Yildirim Uslu et al. was limited to 1 week, primarily reflecting the short-term analgesic effect of the local anesthetic rather than the longer-term effect of the corticosteroid administered in our cohort. Second, they used prilocaine, a short-acting local anesthetic, whereas ropivacaine was used in the present study. Ropivacaine has a longer duration of action and a more favorable sensory-to-motor differential block profile, characteristics that may influence early clinical outcomes, although their impact on longer-term pain relief remains uncertain (23–25).

### Implications for patient management pathways

Beyond the clinical outcomes observed with individual procedures, the principal contribution of this study is the evaluation of an IRU as a potential model of care for patients with refractory musculoskeletal pain. Before the establishment of the IRU, the standard pathway for patients with myofascial pain syndromes, axial pain, or osteoporotic vertebral fractures at our institution was largely limited to optimization of pharmacological treatment according to the WHO analgesic ladder, followed by referral to other specialties, predominantly the Pain Unit, Rehabilitation, or Orthopedic Surgery.

This previous care pathway was reflected in the observation that more than 96% of patients undergoing myofascial or ESP blocks had not received any previous infiltration before referral to our unit, despite having clinical indications for these procedures.

Taken altogether, these observations suggest that the establishment of the IRU expanded access to ultrasound-guided interventional procedures within the rheumatology department for patients who had not received these treatments, despite having clinical indications for them.

The implementation of the IRU may have modified the clinical management pathway for patients with refractory musculoskeletal pain. Instead of being routinely referred to other specialties, approximately 70% of patients were discharged directly from the unit, most commonly because of clinical improvement. Nevertheless, nearly 30% of patients still required referral to another specialty, likely reflecting the persistent complexity of chronic musculoskeletal pain and the multidisciplinary management often required for these patients (26).

This model of care may have implications for healthcare efficiency and resource utilization, although these potential system-level effects could not be formally evaluated within the design of the present study.

The differential referral pattern observed across procedural groups also provides clinically relevant information for discharge planning. Patients undergoing shoulder procedures were referred predominantly to Rehabilitation, likely reflecting the need for functional recovery programs following structural interventions (17). In contrast, patients in the myofascial infiltration and ESP block groups were more frequently referred to the Pain Unit, consistent with the greater chronicity and central sensitization commonly associated with these conditions (27). These findings may help inform the development of integrated care pathways between interventional rheumatology and other specialties. Nevertheless, the absence of a historical comparator precludes definitive conclusions regarding the impact of the IRU on referral patterns and healthcare resource utilization.

### Safety profile and feasibility

The safety profile observed in this study was reassuring. Only two adverse events (1.2%) were recorded, both consisting of subcutaneous atrophy secondary to corticosteroid injection, a well-recognized and generally self-limiting complication of depot corticosteroid use. No serious adverse events, including pneumothorax, intravascular injection, or nerve injury, were observed among the 170 procedures performed, including technically demanding interventions such as the ESP blocks and deep myofascial infiltrations.

Although these findings should be interpreted considering the study design and sample size, they support the safety of ultrasound-guided interventional procedures when performed by rheumatologists with specific training in musculoskeletal ultrasound and interventional techniques. This is consistent with the low complication rates reported in previous studies of ultrasound-guided interventional rheumatology procedures (14, 28).

### Educational value and sustainability of the model

As a university-affiliated teaching hospital, the IRU also provides a structured environment for training medical students and rheumatology trainees in musculoskeletal ultrasound and ultrasound-guided interventions.

The incorporation of interventional techniques into rheumatology training programs may be important for the long-term sustainability of this model of care by ensuring that future rheumatologists acquire the expertise required to meet the growing demand for minimally invasive management of musculoskeletal pain (29–31).

### Limitations

This study has several limitations that should be considered. First, the heterogeneous study population, which included multiple procedure techniques and underlying diagnoses, limits the interpretation of pooled effectiveness estimates and precludes procedure-specific conclusions in smaller subgroups. In addition, patients were referred to an IRU, introducing the potential for referral bias and limiting the generalizability of the findings to other clinical settings.

The retrospective, single-center design without control group precludes causal inference. Moreover, follow-up assessments were not standardized, resulting in substantial interindividual variability in the timing of outcome assessment. This represents a potential source of bias, as patients evaluated earlier may have been assessed during the peak treatment response period, whereas those evaluated later may have been assessed after partial attenuation of treatment effect. Although a *post hoc* analysis did not demonstrate a statistically significant differences in NRS reduction or clinically meaningful pain reduction rates across follow-up intervals, the study was not powered to detect such differences, and the small sample size in some intervals limits the precision of these exploratory comparisons.

The operators’ learning curve was not formally evaluated. Although both rheumatologists had received dedicated prior training in interventional rheumatology, ultrasound-guided procedures are inherently operator-dependent, and differences in technical expertise, even within a specialized unit, may have contributed to variability in clinical outcomes.

Functional outcomes and health-related quality of life were not assessed. The inclusion of these patient-centered outcomes would have provided a more comprehensive evaluation of the clinical effectiveness of these interventions.

Finally, no sensitivity analyses were performed given the descriptive and exploratory nature of the study.

## Conclusion

Ultrasound-guided minimally invasive procedures performed within an IRU were associated with high rates of clinically meaningful pain reduction, and a favorable safety profile in patients with chronic musculoskeletal pain. These findings suggest that IRUs may have a role within multidisciplinary pain management pathways, although prospective controlled studies are needed to confirm these findings.

## Data Availability

The data underlying this article are available upon reasonable request to the corresponding author.
